# The Amsterdam wrist rules: the multicenter prospective derivation and external validation of a clinical decision rule for the use of radiography in acute wrist trauma

**DOI:** 10.1186/s12891-015-0829-2

**Published:** 2015-12-18

**Authors:** Monique M. J. Walenkamp, Abdelali Bentohami, Annelie Slaar, M. Suzan H. Beerekamp, Mario Maas, L. Cara Jager, Nico L. Sosef, Romuald van Velde, Jan M. Ultee, Ewout W. Steyerberg, J. Carel Goslings, Niels W. L. Schep

**Affiliations:** Department of Surgery, Trauma Unit, Academic Medical Center, University of Amsterdam, P.O. Box 22660, Amsterdam, 1100 DD The Netherlands; Department of Radiology, Academic Medical Center, University of Amsterdam, P.O. Box 22660, Amsterdam, 1100 DD The Netherlands; Emergency Department, Academic Medical Center, University of Amsterdam, P.O. Box 22660, Amsterdam, 1100 DD The Netherlands; Spaarne Hospital, Department of Surgery, P.O. Box 770, Hoofddorp, 2130 AT The Netherlands; Tergooi Hospitals, Department of Surgery, P.O. Box 10016, Hilversum, 1201 DA The Netherlands; Sint Lucas Andreas Hospital, Department of Surgery, P.O. box 9243, Amsterdam, 1006 AE The Netherlands; Department of Public Health, Erasmus Medical Center, University Medical Center Rotterdam, P.O. Box 2040, Rotterdam, 3000 CA The Netherlands; Maasstad Hospital, Department of Surgery, P.O. Box 9100, Rotterdam, 3007 AC The Netherlands

**Keywords:** Wrist injury, Wrist trauma, Distal radius fractures, Distal ulna fractures, Wrist fractures, Carpal bone fractures, Clinical decision rule, Clinical decision model, Derivation, External validation, Amsterdam wrist rules

## Abstract

**Background:**

Although only 39 % of patients with wrist trauma have sustained a fracture, the majority of patients is routinely referred for radiography. The purpose of this study was to derive and externally validate a clinical decision rule that selects patients with acute wrist trauma in the Emergency Department (ED) for radiography.

**Methods:**

This multicenter prospective study consisted of three components: (1) derivation of a clinical prediction model for detecting wrist fractures in patients following wrist trauma; (2) external validation of this model; and (3) design of a clinical decision rule. The study was conducted in the EDs of five Dutch hospitals: one academic hospital (derivation cohort) and four regional hospitals (external validation cohort). We included all adult patients with acute wrist trauma. The main outcome was fracture of the wrist (distal radius, distal ulna or carpal bones) diagnosed on conventional X-rays.

**Results:**

A total of 882 patients were analyzed; 487 in the derivation cohort and 395 in the validation cohort. We derived a clinical prediction model with eight variables: age; sex, swelling of the wrist; swelling of the anatomical snuffbox, visible deformation; distal radius tender to palpation; pain on radial deviation and painful axial compression of the thumb. The Area Under the Curve at external validation of this model was 0.81 (95 % CI: 0.77–0.85). The sensitivity and specificity of the Amsterdam Wrist Rules (AWR) in the external validation cohort were 98 % (95 % CI: 95–99 %) and 21 % (95 % CI: 15 %–28). The negative predictive value was 90 % (95 % CI: 81–99 %).

**Conclusions:**

The Amsterdam Wrist Rules is a clinical prediction rule with a high sensitivity and negative predictive value for fractures of the wrist. Although external validation showed low specificity and 100 % sensitivity could not be achieved, the Amsterdam Wrist Rules can provide physicians in the Emergency Department with a useful screening tool to select patients with acute wrist trauma for radiography. The upcoming implementation study will further reveal the impact of the Amsterdam Wrist Rules on the anticipated reduction of X-rays requested, missed fractures, Emergency Department waiting times and health care costs.

**Trial registration:**

This study was registered in the Dutch Trial Registry, reference number NTR2544 on October 1^st^, 2010.

**Electronic supplementary material:**

The online version of this article (doi:10.1186/s12891-015-0829-2) contains supplementary material, which is available to authorized users.

## Background

Wrist trauma is one of the most common Emergency Department (ED) attendances and accounts for approximately 20 % of all injuries [1–3]. Only 39 % of patients with wrist trauma have a fracture; however, most patients are routinely referred for radiography [4–6].

Unlike ankle [7], elbow [8] and knee [9] injury, there are no guidelines or criteria available that indicate which patients with wrist trauma require an X-ray. A clinical decision rule that selects patients for radiography could avoid unnecessary wrist X-rays and therefore decrease radiation exposure; ED waiting times and reduce health care expenditure [5, 10–12].

Two previous studies investigated the diagnostic value of physicals findings in patients with acute wrist trauma [13, 14]. However, these studies were limited by small study populations and did not present a clinical decision rule.

The purpose of this study was to derive and externally validate a clinical decision rule that selects patients with acute wrist trauma in the Emergency Department for radiography.

## Methods

### Study design and setting

The study protocol has previously been published [15]. We performed a multicenter prospective study that consisted of three components: (1) derivation of a clinical prediction model for detecting wrist fractures in patients following wrist trauma; (2) external validation of the model in a new patient population enrolled in a different setting; and (3) design of a clinical decision rule. The study was conducted in the Emergency Departments of five Dutch hospitals from November 11, 2010 to June 25 2014. The participating hospitals included one academic hospital and four regional teaching hospitals: the Academic Medical Centre, the Tergooi Hospital, the Sint Lucas Andreas Hospital, the Flevo Hospital and Spaarne Hospital. The central Medical Ethical Review Committee of the Academic Medical Center approved the study without the need for informed consent (NL34430.018.10). Additionally, the local Medical Ethical Review Committees of all four participating hospitals approved the study.

The derivation cohort comprised all patients enrolled in the academic hospital. The validation cohort included all patients enrolled in the four other participating hospitals.

### Selection of participants

We included all consecutive adult patients who presented to the Emergency Department with pain or tenderness secondary to wrist trauma. The wrist was defined as the proximal segment of the hand, including the carpal bones and the associated soft parts; and the distal segment of the ulnar and radial bone. Wrist trauma was defined as any high or low energetic trauma involving the wrist, such as a fall on outstretched hand (FOOSH). We excluded patients whose injury occurred more than 72 h previously or multi trauma patients (Injury Severity Score ≥16). Patients who already had an X-ray made previous to their visit to the Emergency Department (for example requested by their general practitioner or by another hospital) were excluded as well. Additionally, physicians were instructed not to include patients if radiographs had already been ordered and they were aware of the outcome (fracture present or not).

### Data collection and variables

Eligible patients were included upon presentation in the Emergency Department. Data were collected prospectively by the treating physicians on standardized Case Record Forms (CRF). Patients were evaluated for 19 clinical variables including patient characteristics, physical examination and functional testing (Table 1). We based the selection of variables on clinical experience and previous studies [13, 14]. The questions on the Case Record Form (CRF) were presented in a dichotomous nature (yes/no). Eligibility and data collection forms were verified by two authors by cross-checking the medical records of all patients six months after inclusion.

**Table 1:** Potential predictors considered in the full model

Sex (if male)	
Age (continuous)	
Swelling of wrist	
Swelling of the anatomical snuffbox	
Visible deformation	
Distal radius tender to palpation	
Distal ulna tender to palpation	
Anatomical snuffbox tender to palpation	
Scaphoid tubercle tender to palpation	
Active mobility painful	
dorsiflexion	
palmar flexion	
supination	
pronation	
ulnar deviation	
radial deviation	
Functional tests painful	
radioulnar ballottement test^a^	
axial compression of forearm	
axial compression thumb	
pinch grip test	

^a^Test is positive if pain occurs when the ulna is translated from volar to dorsal while the radius manually fixated Except for age, all predictors were ordinal and coded yes (if present) or no (of not present)

The assessors were all physicians and included consultant emergency medicine physicians; emergency medicine residents; surgical residents; orthopaedic residents and general practice residents. All physicians received regular instructions and training on how to assess the clinical variables in a standardized manner. Additionally, we provided informative pocket cards and posters. In order not to disrupt common practice, referral for radiography and type of treatment were at the discretion of the treating physician. Although the study did not mandate radiographs on all wrist-injured patients, only 5 out of 1019 patients (0.5 %) did not receive an X-ray of the wrist.

### Outcomes

The reference standard was the presence of a fracture of the distal radius, ulna or one of the carpal bones, as assessed by the attending radiologist on the X-ray at presentation. A fracture was defined as a disruption of one or more of the cortices. A fissure and an avulsion were recorded as a fracture. The radiologist was blinded to the contents of the Case Record Forms. Radiographic series comprised at least one posterior-anterior (PA) and one lateral view with 90 degrees of elbow flexion; and any further conventional imaging available (for example scaphoid series). We did not take findings on additional Computed Tomography scans or Magnetic Resonance Image scans into account.

### Sample size

A common rule of thumb to determine the sample size of the development of a prediction model is at least ten events (fractures) per variable [16]. Patients were evaluated for 19 variables. Therefore the inclusion of minimum of 190 patients who sustained a fracture was required in the derivation cohort. According to a similar rule of thumb, external validation requires at least 100 patients with an event (fracture) and 100 patients without an event (no fracture) [16]. We continued enrolling patients after the required sample size was achieved to maintain the study infrastructure required for the subsequent implementation study.

### Analysis

For efficient statistical analysis [17–19], we used imputation techniques to impute the missing values (*aregImpute* function from the *Hmisc* library, R, version 3.0.1.) For each variable containing missings, the *aregImpute* package draws values from a random sample from the non-missing values with replacement. Using this data, aregImpute fits a flexible model that predicts the missing target variable while finding its optimum transformation. Each missing variable is then imputed with the observed value whose predicted transformed value is closest to the predicted transformed value of the missing variable. We considered an imputation model that included all dichotomous variables; prehensile grip strength and the outcome. The set of first imputations was used for the analyses.

### Model development and internal validation

We derived two clinical prediction models: one for all wrist fractures (distal ulna, distal radius and carpal bone) and one for distal radius fractures only. Using data on patients enrolled in the academic hospital, multivariate logistic regression models with all 19 potential predictors were fit. These full modes were reduced using a stepwise backward elimination process based on a liberal p-value of 0.2 [20]. To estimate the internal validation of performance we used bootstrapping (500 replications). Bootstrapping provided the shrinkage factor that was used for the regression coefficients [21].

### External model validation and final model development

To assess general applicability, we validated the shrunk models in the cohort that included all patients enrolled in the four other participating hospitals. For each patient in the validation cohort, the probability of a wrist fracture or of a distal radius fracture was calculated using the prediction models. The validity of the models was assessed by comparing the predicted probabilities of a fracture with the observed fractures. To estimate the ability of the models to discriminate between patients with and without a fracture, we calculated the Areas under the Receiver Operating Characteristics Curve (AUC). The AUC ranges from 0.5–1, with a higher score indicating more accurate predictions. The models were also evaluated for their agreement between predicted fractures and observed fractures. This is otherwise known as the model calibration and was assessed by plotting the predicted probability of a fracture and the observed frequency of fractures. The ideal slope of such a plot is 1, indicating perfect agreement between observed and predicted risks [20]. As a final step, the models were fit on data from both cohorts combined to obtain robust estimates of the regression coefficients. These final modes were internally validated by bootstrapping as for the initial models.

### Clinical decision rule

A clinical prediction model provides an estimated risk of a certain outcome. A clinical decision *rule* goes one step further and links a recommendation to the predicted risk. In this study, the recommendation would be to request an X-ray yes or no. A clinical decision rule therefore requires a cut-off value for the predicted probability of a fracture to classify patients as low or high risk (or recommend an X-ray yes or no). We decided beforehand to select a cut-off value at which the sensitivity of the Amsterdam Wrist Rules would not drop below 98 %, while maintaining the highest specificity.

## Results

### Characteristics of study subjects

**Fig. 1:**
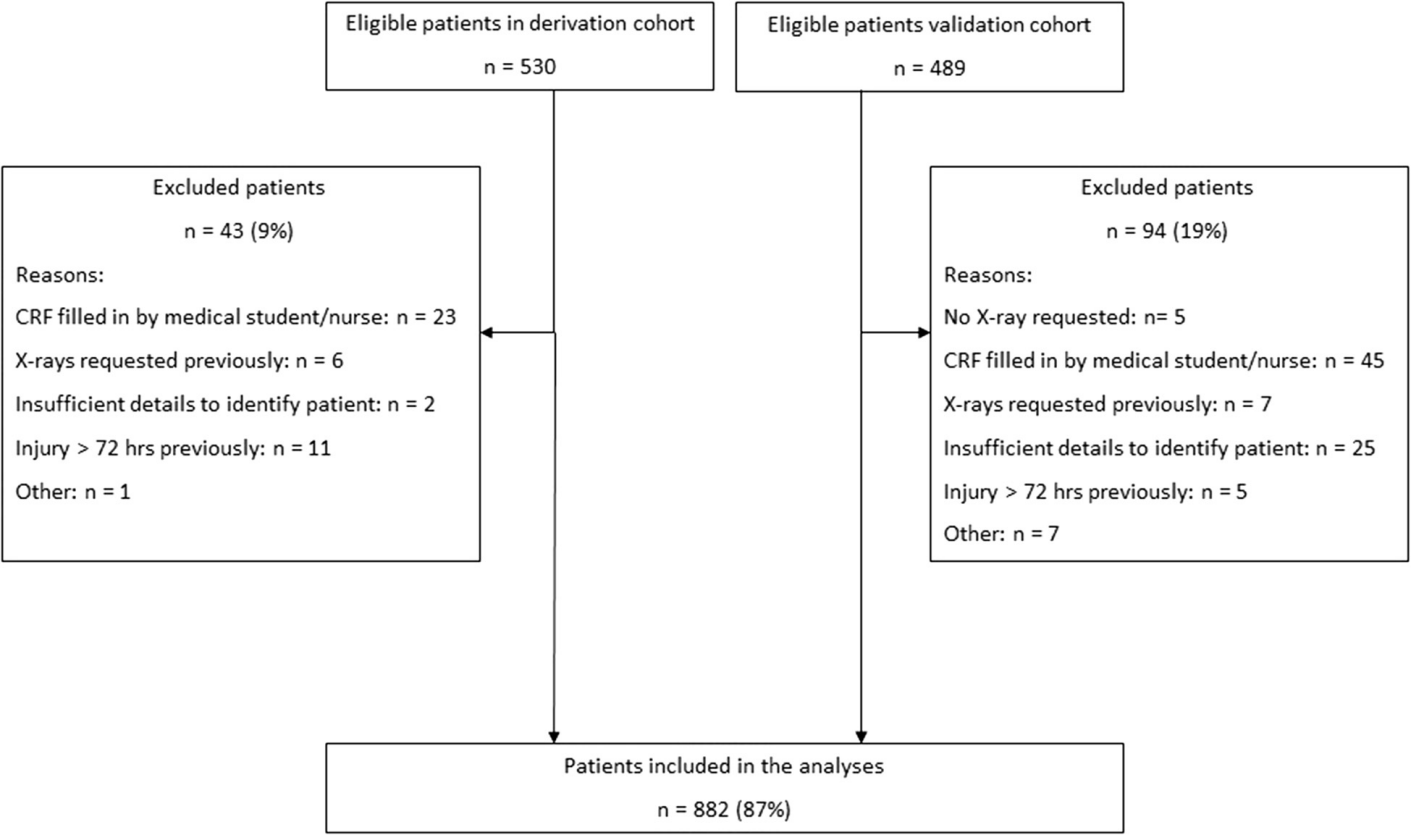
Flowchart

We enrolled 1019 patients from five participating hospitals. A total of 137 patients (13 %) were excluded patients from the analysis for various reasons (Fig. 1). In total, 882 patients were analyzed (Table 2). In 470 patients (53 %), a fracture of the distal radius, distal ulna or one of the carpal bones was identified on conventional radiographic series. A distal radius fracture was the most common fracture (44 %).

**Table 2:** Clinical and demographic Characteristics of derivation cohort and validation cohort

Characteristics	Derivation cohort^a^ (*n* = 487)	Validation cohort^b^ (*n* = 395)	Total^c^ (*n* = 882)
Age, median (IQR)	48 (29–61)	52 (33–68)	50 (31–63)
Female, No. (%)	276 (57)	256 (64.8)	532 (60.3)
Mechanism of injury, No. (%)			
FOOSH	320 (65.7)	265 (67.1)	585 (66.3)
Direct blow or compression	42 (8.6)	22 (5.5)	64 (7.3)
Traffic accident	37 (7.6)	33 (8.4)	70 (8.0)
Forced hyperflexion	19 (3.9)	6 (1.5)	25 (2.8)
Punch	13 (2.7)	4 (1.0)	17 (1.9)
Other/unknown	56 (11.5)	65 (16.5)	121 (13.7)
Patients with a wrist fracture^d^, No. (%)	251 (51.5)	219 (55.4)	470 (53.3)
Distal radius fracture, No. (%)^e^	200 (41.1)	184 (46.6)	384 (43.5)
Triquetrum fracture, No. (%)^e^	26 (5.3)	11 (2.8)	37 (4.2)
Scaphoid fracture, No. (%)^e^	25 (5.1)	23 (5.8)	48 (5.4)
Isolated distal ulna fracture, No. (%)^e^	7 (1.4)	3 (0.8)	10 (1.1)
Other carpal bone fracture, No. (%)^e^	2 (0.4)	1 (0.3)	3 (0.3)
Patients with multiple wrist fractures, No. (%)	7 (1.4)	4 (1)	11 (1.2)
Treatment^f^			
Expectant	38 (7.8)	28 (7.0)	66 (7.5)
Compression bandage	94 (19.3)	73 (18.5)	167 (18.9)
Plaster immobilisation	243 (49.9)	190 (48.1)	433 (49.1)
Reduction and plaster immobilisation	94 (19.3)	82 (20.8)	176 (19.9)
Primary operative	18 (3.7)	17 (4.3)	35 (4.0)
Unknown^g^	0	5 (1.3)	5 (0.6)

Abbreviations: *IQR* interquartile range, *FOOSH* fall on outstretched hand

^a^Data from the academic hospital, the derivation cohort

^b^Data from the other four hospitals, the validation cohort

^c^The final derivation cohort

^d^Fracture of the distal radius, distal ulna or one of the carpal bones

^e^Percentage of total number of patients. Because some patients had multiple fractures, the total number of different fractures is not equal to number of patients with a wrist fracture

^f^Treatment for patients with and without fractures

^g^Not recorded in patients files

In the derivation cohort, 487 patients were analyzed with a median age of 48 years (interquartile range, 29 - 61) and women were slightly overrepresented (57 %). A fall on outstretched hand was the most common mechanism of injury (66 %). In 251 patients (52 %) in the derivation cohort, a fracture of the distal radius, ulna or one of the carpal bones was identified.

In the validation cohort, 395 patients with similar demographic characteristics were analyzed (Table 2). In 219 of these patients (55 %), a fracture of the distal radius, distal ulna or one of the carpal bones was identified.

### Missing values and imputation

In both the derivation and the development cohort, around 80 % of the cases had fully complete Case Record Forms. With the exception of prehensile grip strength, missing values comprised less than 5 % for each variable (see Additional file 1).

### Model development

A clinical prediction model for all fractures was derived that included eight variables: age; sex (if male), swelling of the wrist; swelling of the anatomical snuffbox, visible deformation; distal radius tender to palpation; pain on radial deviation and painful axial compression of the thumb. The Area Under the Curve (AUC) of this model was 0.84 (95 % CI: 0.81–0.88) and 0.82 (95 % CI: 0.79–0.85) after correcting for model optimism by bootstrapping.

The coefficient of each dichotomous variable reflects the amount of change in the probability of a fracture (Table 3). The presence of a dichotomous variable with a positive coefficient adds to the probability of a fracture. The presence of a dichotomous variable with a negative coefficient decreases the probability. The coefficient of the continuous variable age reflects the amount of change in probability for every ten-year increase in age. Except for painful axial compression of the thumb (coefficient -0.37), the presence of all variables adds to the probability of a fracture. Painful axial compression of the thumb decreases the probability of a fracture.

**Table 3:** Predictors in model for all fractures^a^

Predictor	Coefficient (95 % CI)	Odds ratio (95 % CI)
Age (per 10 years)	0.35 (0.22–0.49)	1.04 (1.02–1.05)
Sex (if male)	0.38 (–0.10–0.86)	1.46 (0.90–2.35)
Swelling wrist	1.48 (1.00–1.96)	4.40 (2.72–7.11)
Swelling anatomical snuffbox	0.47 (-0.08–1.02)	1.60 (0.92–2.78)
Visible deformation	1.32 (0.54–2.09)	3.73 (1.72–8.11)
Distal radius tender to palpation	0.88 (0.23–1.53)	2.41 (1.25–4.63)
Pain with radial deviation	0.67 (0.08–1.26)	1.95 (1.08–3.51)
Pain with axial compression of the thumb	−0.37 (-0.88–0.14)	0.69 (0.41–1.15)

The coefficient of each dichotomous variable reflects the amount of change in the log odds of a fracture. The coefficient of the continuous variable age reflects the amount of change in the log odds of a fracture for every ten-year increase in age

Abbreviations: *CI* Confidence Interval

^a^Derived from data from the academic hospital

A clinical prediction model for only distal radius fractures was derived that also included eight variables: age; swelling of the wrist; visible deformation; distal radius tender to palpation; pain on ulnar deviation; palmar flexion, supination and the painful radioulnar ballottement test (Table 4). The presence of all variables except pain on ulnar deviation increases the probability of a distal radius fracture. Pain on ulnar deviation (coefficient -0.67 (95 % CI: -1.35–0.02) decreases the probability of a distal radius fracture. The Area Under the Curve (AUC) of this model was 0.91 (95 % CI: 0.88–0.93) and 0.90 (95 % CI: 0.87–0.92) after optimism correction by bootstrapping.

**Table 4:** Predictors in model for distal radius fractures^a^

Predictor	Coefficient (95 % CI)	Odds ratio (95 % CI)
Age (per 10 years)	0.40 (0.25–0.54)	1.04 (1.02–1.06)
Swelling wrist	2.07 (1.44–2.70)	7.92 (4.24–14.8)
Visible deformation	1.38 (0.59–2.17)	3.97 (1.81–8.74)
Distal radius tender to palpation	2.75 (1.22–4.28)	15.7 (3.40–72.4)
Pain on palmar flexion	0.64 (-0.15–1.43)	1.90 (0.86–4.18)
Pain on supination	0.81 (0.15–1.47)	2.25 (1.16–4.37)
Pain on ulnar deviation	−0.67 (-1.35–0.02)	0.51 (0.26–1.02)
Pain on radioulnar ballottement test	0.56 (-0.02–1.15)	1.76 (0.98–3.16)

The coefficient of each dichotomous variable reflects the amount of change in the log odds of a fracture. The coefficient of the continuous variable age reflects the amount of change in the log odds of a fracture for every ten-year increase in age

Abbreviations: *CI* Confidence Interval

^a^Derived from data from the academic hospital

### External model validation and test characteristics

The external performance of the models was assessed in the 395 patients in the validation cohort. The Area Under the Curve at external validation of the model for all fractures was 0.81 (95 % CI: 0.77–0.85) and the calibration slope was 0.94 (95 % CI: 0.74–1.13). The Area Under the Curve at external validation of the model for only distal radius fractures was 0.86 (95 % CI: 0.82–0.89) and the calibration slope was 1.07 (95 % CI: 0.84–1.29).

The Amsterdam Wrist Rules (AWR) for all wrist fractures showed a sensitivity and specificity of 98 % (95 % CI: 95–99 %) and 21 % (95 % CI: 15–28 %) (Table 5). Its negative predictive value was 90 % (95 % CI: 81–99 %). The sensitivity and specificity for only distal radius fractures were 98 % (95 % CI: 97–100 %) and 25 % (95 % CI: 19–31 %) (Table 5). The AWR was able to rule out 19 % (41 / 219) of the patients without a wrist fracture and 25 % (53 / 211) of the patients without a distal radius fracture. If the AWR had been used for all fractures, an X-ray would have been requested for 89.6 % (354 / 395) of patients instead of 100 %. This is an absolute reduction of 10.4 %. The final formula to calculate the probabilities are depicted in Table 6. The AUC of the final model after bootstrapping was 0.88 (95 % CI: 0.86–0.90)

**Table 5:** The performance of the Amsterdam Wrist Rules at external validation (*N* = 395)

All Fractures		
Amsterdam Wrist Rules indicate X-ray	215	139
Amsterdam Wrist Rules indicate no X-ray	4	37
Total	219	176
Sensitivity (%)	98.2 (95.1–99.4)	
Specificity (%)	21.0 (15.4–27.9)	
Distal Radius Fractures		
Amsterdam Wrist Rules indicate X-ray	179	158
Amsterdam Wrist Rules indicate no X-ray	3	53
Total	184	211
Sensitivity (% [95 % CI])	98.4 (96.5–100.0)	
Specificity (% [95 % CI])	25.1 (19.3–31.0)	

Abbreviations: *CI* Confidence Interval

The cut-off point for X-ray yes or no was a predicted probability of 21 % for all fractures and 4 % for only distal radius fractures

**Table 6:** Calculation of the linear predictor and probability^a^

Linear predictor ALL WRIST FRACTURES
0.0309* age + 0.5862 + (if male) + 1.1486 * (if swelling wrist present) + 0.5757 (if swelling anatomical snuff box is present) + 1.7123 *(if visible deformation present) + 0.7029 * (if distal radius tender to palpation) + 0.4963 *(if pain on radial deviation) + -0.1793 * (if on axial compression thumb) - 3.616
Linear predictor DISTAL RADIUS FRACTURES
0.0341* age + 1.7298 * (if swelling of wrist present) + 1.6462 *(if visible deformation present) + 1.8117 * (if distal radius tender to palpation) + 0.4228 *(if pain on palmar flexion) + 0.6567 * (if pain on supination) – 0.2941 (if pain on ulnar deviation) + 0.5949 * (if pain during radioulnar ballottement test) - 6.0202
Formula to calculate probability of a fracture based on final model
1/ (1 + EXP(-Linear Predictor))

* signifies a multiplication sign

All individual parameters add to the probability of a fracture

^a^Coefficients were derived from a fit of the model on both cohort combined (*N* = 882) and corrected for optimism by bootstrapping (*N* = 500 replications)

## Discussion

We have developed a clinical prediction rule with a high sensitivity (98 %) and negative predictive value (90 %) for fractures of the wrist. Previous studies have illustrated that the X-ray referral policy for patients with wrist trauma is often obscure and unfounded, and to date no guidelines or criteria were available [6, 13, 14, 22]. The Amsterdam Wrist Rules can provide physicians with an externally validated screening tool trauma in the Emergency Department to select patients with acute wrist trauma for radiography.

The foremost strength of the Amsterdam Wrist Rules is that it is one of the few clinical decision rules that have been externally validated. Most clinical decision rules only undergo internal validation, often by bootstrapping [23]. However, evaluating the performance of a prediction model or a clinical decision rule in a new patient population is essential before its implementation. The Amsterdam Wrist Rules underwent this most stringent form of external validation: the rules were tested in a patient population from different type of hospitals with different physicians [24]. The performance of the Amsterdam Wrist Rules expressed in the AUC reflects excellent discriminative ability in a new patient population.

However, the Amsterdam Wrist Rules showed disappointing specificity at external validation. We could have developed the clinical decision rule with higher specificity and number of X-rays avoided. However, this would have resulted in a decreased sensitivity and consequently more fractures missed. Preferably, clinical decision rules in the Emergency Department have a very high sensitivity and negative predictive value. We believe that physicians will be reluctant to use any clinical decision rule with a sensitivity below 98 % [25]. In a similar way Stiell et al. devised the Ottawa Ankle Rules with a sensitivity of 100 % because they felt that physicians would not accept to miss fractures. However, they also expressed the hope that society will come to accept the small price of an occasionally missed fracture that would probably have led to very little morbidity for the patients [7].

If the Amsterdam Wrist Rules had been applied in the external validation cohort, the 10 % absolute reduction in X-rays would have been accompanied by 4 (1.6 %) missed fractures: two scaphoid fractures, one intra-articular distal radius fracture and one extra-articular distal radius fracture. None of these fractures were dislocated or required surgery. Consequently, we advise caution in the use of the Amsterdam Wrist Rules before its true effects on both patient care and use of resources have been evaluated in the upcoming implementation study. After implementation of the Ottawa Ankle Rules, a relative reduction of 26 % of ankle radiographies was recorded in the intervention hospital without any missed fractures or patient discontent [11, 26].

Another difference between the study population of the Ottawa Ankle Rules and our study is the pre-test probability. Ankle fractures occurred in around 14 % of the patients with ankle injury whereas 53 % of our patients had sustained a wrist fracture. This issue was also raised by colleagues van der Brand et al., who concluded that the high percentage of patients that had sustained a fracture warrants radiography in all patients with wrist trauma [6]. We have to agree that the low specificity of the Amsterdam Wrist Rules is somewhat disappointing. However, we feel that referring every patient for radiography would be rash and not appropriate in light of the ever-rising health care costs [22]. Moreover, although specificity of the Amsterdam Wrist Rules was low at external validation, it is better than the current practice to refer nearly all patients for radiography [6, 22]. Furthermore, while a 10 % reduction in X-rays may seem small, on a national level it corresponds to thousands of X-rays annually.

We decided to derive a second decision rule dedicated to the most common wrist fracture: a distal radius fracture. The performance of this model was better and therefore we recommend its use in patients who are only suspected of a distal radius fracture.

We are currently also working on deriving a clinical decision rule dedicated to detecting scaphoid fractures.

**Fig. 2:**
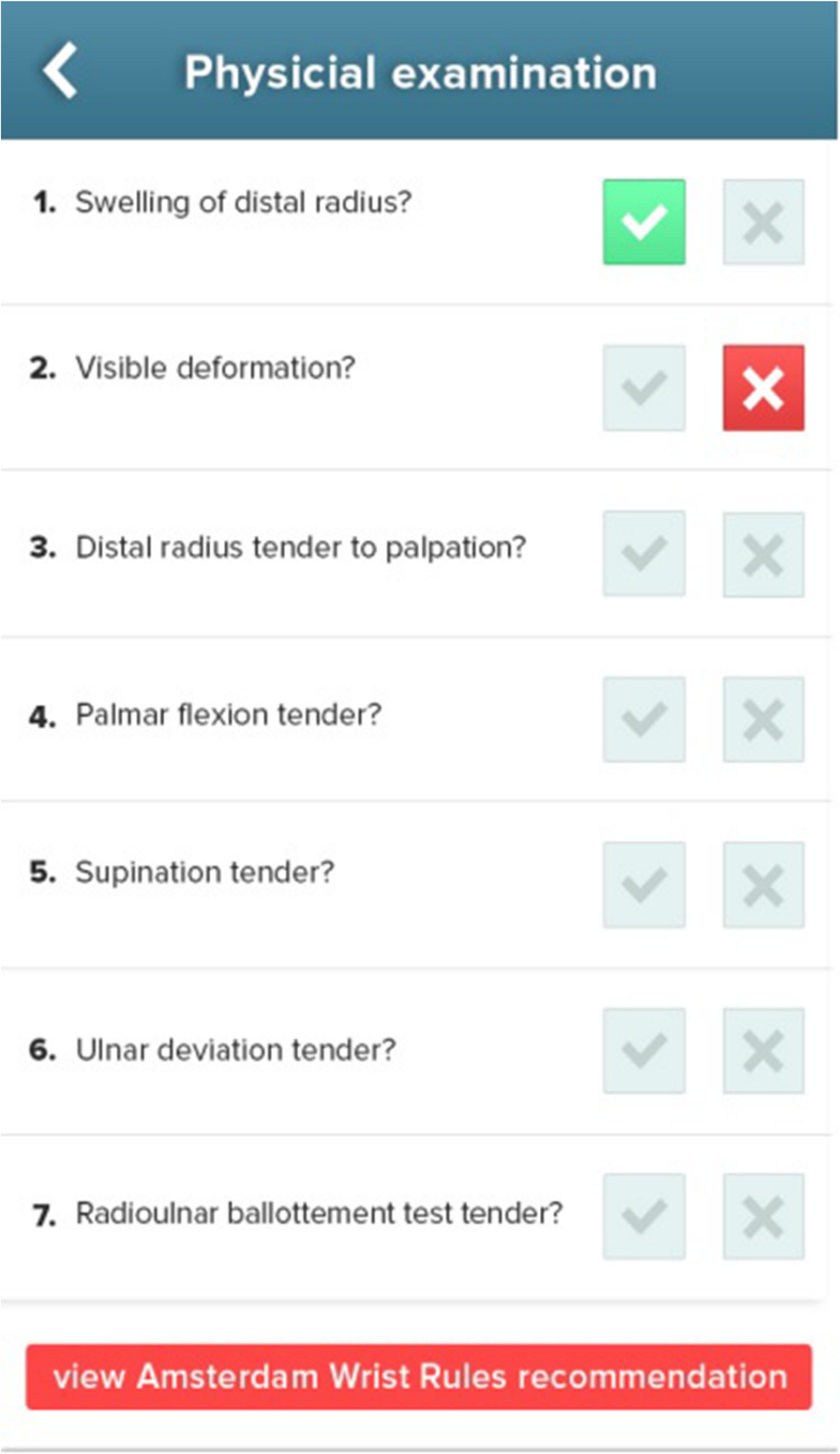
A screen shot of the smart phone application that will be used during the implementation study. After entering the clinical findings, the application will calculate the probability of a distal radius fracture using the formula depicted in Table 1. If the probability of a distal radius fracture is <4 %, the Amsterdam Wrist Rules application will recommend no radiography. The application was built by ApplicationBuilders

To determine the actual effect of the Amsterdam Wrist Rules in clinical practise we have recently started the implementation study and currently enrolled over a 100 patients. In this study, we will evaluate the reduction in radiographs requested, costs, ED waiting times, missed fractures, patient satisfaction and clinical sensibility to physicians. To simplify application of the Amsterdam Wrist Rules, the formula to predict the probability of a fracture (Table 6) will be made available in a smartphone application (Fig. 2). Upon entering the clinical variables, the application will calculate the probability of fracture and give a recommendation (X-ray yes/no). A secondary implementation study is scheduled to take place in general practitioner’s offices. Implementation in this more general setting, where X-ray apparatuses are not readily available, might result in a higher diagnostic yield and even more cost savings.

This study has several limitations. According to methodological standards for the development of clinical decision rules in the Emergency Department, the reliability of predictor variables should be tested by determining the intraobserver and interobserver agreement [25]. However, we considered it unethical to subject patients with a painful wrist to two comprehensive physical examinations. Therefore we were unable to assess the consistency of the candidate predictors.

Ideally, the reference standard for this study was the presence of a distal radius fracture on Multi Slice Computed Tomography (CT) or Magnetic Resonance Imaging (MRI) scans [16]. However, considering the number of participants this was both unethical and not feasible. Therefore, the outcome used for the analysis was the radiographic diagnosis made by the attending independent skeletal radiologist based on the available radiographs at presentation. Consequently, this approach has resulted in a clinical decision rule that does not detect injuries that are not diagnosed on conventional radiography.

## Conclusion

The Amsterdam Wrist Rules is a clinical prediction rule with a high sensitivity and negative predictive value for fractures of the wrist. The Amsterdam Wrist Rules can provide physicians in the Emergency Department with a useful screening tool to select patients with acute wrist trauma for radiography.

## Additional file

Additional file 1.
**Number of patients with missings according to variable and characteristics of patients with and without prehensile grip strength as missing variable.** (DOC 48 kb)

## Abbreviations

AUC: Areas under the Receiver Operating Characteristics Curve
AWR: Amsterdam Wrist Rules
ED: Emergency Department
FOOSH: Fall on outstretched hand
