# Supplementary material for: The Amsterdam wrist rules: the multicenter prospective derivation and external validation of a clinical decision rule for the use of radiography in acute wrist trauma
Source: BMC Musculoskelet Disord. 2015 Dec 18;16:389. doi: 10.1186/s12891-015-0829-2 (PMC4683697; doi:10.1186/s12891-015-0829-2)
Supplement: Additional file 1. — Number of patients with missings according to variable and characteristics of patients with and without prehensile grip strength as missing variable. (DOC 48 kb) [file 12891_2015_829_MOESM1_ESM.doc]

**Supplementary materials**

**Number of patients with missings according to variable**

| **Variables** | **Derivation cohort^a^**  **(n = 487), No. (%)** | **Validation cohort^b^ (n = 395), No. (%)** |
| --- | --- | --- |
| **Complete cases** | **407 (83.6)** | **320 (81.0)** |
| **Age** | **0** | **0** |
| **Sex** | **0** | **0** |
| **Mechanism of injury** | **0** | **0** |
| **Swelling of distal radius** | **1 (0.2)** | **2 (0.5)** |
| **Visible deformation** | **4 (0.8)** | **7 (1.8)** |
| **Distal radius tender to palpation** | **0 (0.6)** | **1 (0.3)** |
| **Dorsiflexion** | **3 (0.6)** | **2 (0.5)** |
| **Palmar flexion** | **3 (0.6)** | **7 (1.8)** |
| **Supination** | **3 (0.6)** | **3 (0.8)** |
| **Pronation** | **3 (0.6)** | **4 (1.0)** |
| **Ulnar deviation** | **3 (0.6)** | **5 (1.3)** |
| **Radial deviation** | **3 (0.6)** | **8 (2.0)** |
| **Radioulnar ballottement test** | **16 (3.3)** | **17 (4.3)** |
| **Axial compression of forearm** | **11 (2.3)** | **14 (3.5)** |
| **Prehensile grip strength** | **55 (11.3)** | **45 (11.4)** |
| **Distal radius fracture (outcome)** | **0** | **0** |

**^a^ Data from the academic hospital.**

^b^ Data from the other four hospitals.

Characteristics of patients with and without prehensile grip strength as missing variable

| **Characteristics** | **Missing (N=100)** | **Non-missing (N=782)** |
| --- | --- | --- |
| Age | 55 (39-68) | 49 (31-63) |
| Female, No. (%) | 59 (59.0) | 473 (60.5) |
| Patients with distal radius fracture, No. (%) | 57 (57.0) | 327 (41.8) |
| Patients with other wrist fracture than distal radius No. (%)^a^ | 13 (13.0) | 73 (9.3) |
| Patients with multiple wrist fractures No. (%)^b^ | 2 (2.0) | 9 (1.2) |
| Complete cases | 92 (92.0) | 753 (96.3) |
| Treatment^c^ |  |  |
| Expectant | 5 (5.0) | 61 (7.8) |
| Compression bandage | 8 (8.0) | 159 (20.3) |
| Plaster immobilisation | 48 (48.0) | 385 (49.2) |
| Reduction and plaster immobilisation | 30 (30.0) | 146 (18.7) |
| Primary operative | 9 (9.0) | 26 (3.4) |
| Unknown^d^ | 0 | 5 (0.6) |

^a^Patient without a distal radius fracture but with an isolated fracture of the ulna or one of the carpal bones.

^b^Patients that sustained a fracture of distal radius and one of the carpal bones.

^c^Patients with and without fractures

^d^Not recorded in patients files
